# Supplementary material for: Inferring neuron-neuron communications from single-cell transcriptomics through NeuronChat
Source: Nat Commun. 2023 Feb 28;14:1128. doi: 10.1038/s41467-023-36800-w (PMC9974942; doi:10.1038/s41467-023-36800-w)
Supplement: Supplementary file 3 — Reporting Summary [file 41467_2023_36800_MOESM3_ESM.pdf]

nature portfolio

Corresponding author(s): Qing Nie

Last updated by author(s): Feb 5, 2023

Reporting Summary

Nature Portfolio wishes to improve the reproducibility of the work that we publish. This form provides structure for consistency and transparency in reporting. For further information on Nature Portfolio policies, see our Editorial Policies and the Editorial Policy Checklist.

Statistics

For all statistical analyses, confirm that the following items are present in the figure legend, table legend, main text, or Methods section.

n/a	Confirmed	
☐	☒	The exact sample size (*n*) for each experimental group/condition, given as a discrete number and unit of measurement
☐	☒	A statement on whether measurements were taken from distinct samples or whether the same sample was measured repeatedly
☐	☒	The statistical test(s) used AND whether they are one- or two-sided<br>*Only common tests should be described solely by name; describe more complex techniques in the Methods section.*
☒	☐	A description of all covariates tested
☐	☒	A description of any assumptions or corrections, such as tests of normality and adjustment for multiple comparisons
☐	☒	A full description of the statistical parameters including central tendency (e.g. means) or other basic estimates (e.g. regression coefficient) AND variation (e.g. standard deviation) or associated estimates of uncertainty (e.g. confidence intervals)
☐	☒	For null hypothesis testing, the test statistic (e.g. *F*, *t*, *r*) with confidence intervals, effect sizes, degrees of freedom and *P* value noted<br>*Give P values as exact values whenever suitable.*
☒	☐	For Bayesian analysis, information on the choice of priors and Markov chain Monte Carlo settings
☒	☐	For hierarchical and complex designs, identification of the appropriate level for tests and full reporting of outcomes
☐	☒	Estimates of effect sizes (e.g. Cohen's *d*, Pearson's *r*), indicating how they were calculated

Our web collection on statistics for biologists contains articles on many of the points above.

Software and code

Policy information about availability of computer code

Data collection	All data analyzed within this manuscript are publicly available. No additional software was used for the data collection process
Data analysis	We performed the data analysis with newly developed R package NeuronChat, which is available at https://github.com/Wei-BioMath/NeuronChat. NeuronChat is tested on MacOS Big Sur 11.5.1, and its dependencies include R (4.1.1), data.table (1.14.2), dplyr (1.0.9), CellChat (1.1.3), NMF (0.23.0), Seurat (4.1.0), SeuratObject (4.1.0), circlize (0.4.14), ComplexHeatmap (2.8.0), igraph (1.3.4), ggalluvial (0.12.3) and ggplot2 (3.3.6). NeuronChat is released under the GNU General Public License version 3. The code used to produce the analysis in this paper is available at https://github.com/Wei-BioMath/NeuronChatAnalysis2022. scFEA (v1.1-beta0.1) (https://github.com/changwn/scFEA) is used in the benchmarking analysis.

For manuscripts utilizing custom algorithms or software that are central to the research but not yet described in published literature, software must be made available to editors and reviewers. We strongly encourage code deposition in a community repository (e.g. GitHub). See the Nature Portfolio guidelines for submitting code & software for further information.

Data

Policy information about availability of data

All manuscripts must include a data availability statement. This statement should provide the following information, where applicable:

- Accession codes, unique identifiers, or web links for publicly available datasets
- A description of any restrictions on data availability
- For clinical datasets or third party data, please ensure that the statement adheres to our policy

All the single-cell RNA-seq datasets and spatial transcriptomic datasets used are publicly available and from previous publications, and no new experimental data is generated in this study. The mouse datasets analyzed in this study are available from the Gene Expression Omnibus (GEO) repository under the following accession numbers: GSE115746 and GSE185862. The human datasets analyzed in this study are available at https://autism.cells.ucsc.edu. The processed seqFISH+ data are available at https://rubd.github.io/Giotto_site/articles/mouse_seqFISH_cortex_200914.html. The MERFISH dataset is available at https://doi.org/10.35077/g.21. The Visium dataset is available at https://support.10xgenomics.com/spatial-gene-expression/datasets/1.1.0/V1_Adult_Mouse_Brain. NeuronChatDB is included in the NeuronChat repository (https://github.com/Wei-BioMath/NeuronChat), and can be also accessed in table formats (https://github.com/Wei-BioMath/NeuronChatAnalysis2022/tree/main/NeuronChatDB_table). KEGG pathway database is available at https://www.genome.jp/kegg/pathway.html. IUPHAR/BPS Guide to PHARMACOLOGY database is available at https://www.guidetopharmacology.org.

Human research participants

Policy information about studies involving human research participants and Sex and Gender in Research.

Reporting on sex and gender	N/A. All human datasets used are publicly available and from previous publications, and no new experimental data is generated in this study.
Population characteristics	N/A. All human datasets used are publicly available and from previous publications, and no new experimental data is generated in this study.
Recruitment	N/A. All human datasets used are publicly available and from previous publications, and no new experimental data is generated in this study.
Ethics oversight	N/A. All human datasets used are publicly available and from previous publications, and no new experimental data is generated in this study.

Note that full information on the approval of the study protocol must also be provided in the manuscript.

Field-specific reporting

Please select the one below that is the best fit for your research. If you are not sure, read the appropriate sections before making your selection.

☒ Life sciences ☐ Behavioural & social sciences ☐ Ecological, evolutionary & environmental sciences

For a reference copy of the document with all sections, see nature.com/documents/nr-reporting-summary-flat.pdf

Life sciences study design

All studies must disclose on these points even when the disclosure is negative.

Sample size	No new experimental data was generated in this study and no sample size computation was performed. All datasets used in this study were publicly available from previous publications, and used to demonstrate the functionalities of our newly developed package NeuronChat. The datasets included single-cell RNA-seq data from both mouse and human and spatial transcriptomics from different sequencing technologies. Therefore, it is sufficient to demonstrate the functionalities of NeuronChat.
Data exclusions	No biological experiment was conducted in this study and no data were excluded from the analyses on the publicly available data.
Replication	No biological experiment was conducted in this study. For computational task, we repeated the programs independently for different runs and the results are reproducible. The code used to reproduce the results in this study is available at https://github.com/Wei-BioMath/NeuronChatAnalysis2022.
Randomization	No biological experiment was conducted in this study and randomization was not relevant for computational tasks in our study.
Blinding	No biological experiment was conducted in this study and blinding was not relevant for computational tasks in our study.

Reporting for specific materials, systems and methods

We require information from authors about some types of materials, experimental systems and methods used in many studies. Here, indicate whether each material, system or method listed is relevant to your study. If you are not sure if a list item applies to your research, read the appropriate section before selecting a response.

Materials & experimental systems

n/a	Involved in the study
☒	☐ Antibodies
☒	☐ Eukaryotic cell lines
☒	☐ Palaeontology and archaeology
☒	☐ Animals and other organisms
☒	☐ Clinical data
☒	☐ Dual use research of concern

Methods

n/a	Involved in the study
☒	☐ ChIP-seq
☒	☐ Flow cytometry
☒	☐ MRI-based neuroimaging
